# *De novo *454 sequencing of barcoded BAC pools for comprehensive gene survey and genome analysis in the complex genome of barley

**DOI:** 10.1186/1471-2164-10-547

**Published:** 2009-11-20

**Authors:** Burkhard Steuernagel, Stefan Taudien, Heidrun Gundlach, Michael Seidel, Ruvini Ariyadasa, Daniela Schulte, Andreas Petzold, Marius Felder, Andreas Graner, Uwe Scholz, Klaus FX Mayer, Matthias Platzer, Nils Stein

**Affiliations:** 1 Leibniz Institute of Plant Genetics and Crop Plant Research (IPK), Correnstr. 3, D-06466 Gatersleben, Germany; 2 Leibniz Institute for Age Research, Fritz Lipmann Institute (FLI), Beutenbergstr. 11, D-07745 Jena, Germany; 3 MIPS/IBIS, Helmholtz Zentrum München, German Research Center for Environmental Health (GmbH), Ingolstädter Landstr. 1, D-85764 Neuherberg, Germany

## Abstract

**Background:**

*De novo *sequencing the entire genome of a large complex plant genome like the one of barley (*Hordeum vulgare *L.) is a major challenge both in terms of experimental feasibility and costs. The emergence and breathtaking progress of next generation sequencing technologies has put this goal into focus and a clone based strategy combined with the 454/Roche technology is conceivable.

**Results:**

To test the feasibility, we sequenced 91 barcoded, pooled, gene containing barley BACs using the GS FLX platform and assembled the sequences under iterative change of parameters. The BAC assemblies were characterized by N50 of ~50 kb (N80 ~31 kb, N90 ~21 kb) and a Q40 of 94%. For ~80% of the clones, the best assemblies consisted of less than 10 contigs at 24-fold mean sequence coverage. Moreover we show that gene containing regions seem to assemble completely and uninterrupted thus making the approach suitable for detecting complete and positionally anchored genes.

By comparing the assemblies of four clones to their complete reference sequences generated by the Sanger method, we evaluated the distribution, quality and representativeness of the 454 sequences as well as the consistency and reliability of the assemblies.

**Conclusion:**

The described multiplex 454 sequencing of barcoded BACs leads to sequence consensi highly representative for the clones. Assemblies are correct for the majority of contigs. Though the resolution of complex repetitive structures requires additional experimental efforts, our approach paves the way for a clone based strategy of sequencing the barley genome.

## Background

Barley (*Hordeum vulgare *L.) is among the four most important cereal crops worldwide [1]. But in contrast to its agronomical importance efficient gene isolation and genome-wide studies on genetic diversity are hampered by the lack of a reference genome sequence. Such a reference would resolve barley's genetic outfit and would serve as the essential basis to elucidate mechanisms underlying phenotype and traits as well as processes towards plant's adaptation and improvement.

Genome size (~5 Gb) and the high content of repetitive DNA elements (>80%) are the major obstacles towards sequencing the entire barley genome [2,3]. In contrast to Sanger sequencing [4] for a budget of over 100 million USD (T. Sasaki, personal communication) a medium sized plant genome like rice (~400 Mb), the same endeavor for barley was not affordable (for review see [5]). Here, the massively parallel or "next generation sequencing" (NGS) technologies, currently represented by the 454/Roche, Solexa/Illumina and SOLID/ABI platforms, promise to change the situation since several Gigabases (Gb) of sequence data can be accumulated in a few weeks for only a fraction of the costs of Sanger sequencing (for review see [6-8]). NGS technology was successfully applied to *de novo *and re-sequencing of entire prokaryotic genomes [9] and to re-sequencing higher eukaryotes including humans [10-13]. Recently, similar efforts were made in plants by using the Solexa/Illumina platform for re-sequencing of *Arabidopsis thaliana *[14] and by a mixed Sanger and 454/Roche sequencing strategy for grapevine (*Vitis vinifera*) [15]. Whereas the relatively short read lengths of the Solexa/Illumina (GAI/II) and ABI (SOLID) platforms (35-75 and 30-50 bp, respectively) may not yet match the requirements to sequence efficiently across long stretches of repetitive DNA in barley, the 454/Roche system (GS FLX) allows to generate average read lengths of ~250 bp (GS FLX) and ~400 bp (GS FLX Titanium) which are potentially more appropriate to achieve the goals of *de novo *sequencing in complex genomes. However, it remains to be proven whether this holds true with regard to the extraordinarily high content of repetitive DNA elements within the barley genome, often forming blocks extending over regions of several 100 kb [16].

Independently of the platform, two different sequencing strategies are widely used. Whole genome shotgun (WGS) sequencing is based on random shearing of whole genomic DNA and is preferentially applied to medium sized genomes with limited amounts of repetitive DNA. For plant genomes, WGS by NGS was so far restricted to re-sequencing purposes if a reference sequence was available (i.e. *Arabidopsis thaliana *[14]) and to *de novo *sequencing (with or without NGS) of small and medium sized genomes like strawberry (<200 Mb per haploid genome) [17,18] and *Sorghum bicolor *(~730 Mb) [19], or with support of non-NGS data (grapevine) [15].

The second, hierarchical shotgun (HS) approach is based on sequencing bacterial artificial chromosomes (BAC) anchored to a physical map ("clone-by-clone" sequencing). This strategy is more costly than WGS but in return is suitable to generate high quality reference sequences even for highly repetitive genomes [5]. The map-based strategy was not only applied to sequencing the human genome but also to plant genomes such as *Arabidopsis *[20], rice [21] and maize [22]. Due to its accuracy and reliability, the "clone-by-clone" strategy was also favored for producing a high-quality reference sequence of the barley genome [2,23].

Previously, it was demonstrated that genes could be assembled into contigs when barley BACs were sequenced by short reads of ~100 bp provided by the earlier 454/Roche platform (GS20) at sequence coverage of ~10 - 20-fold [24]. Similar results were obtained by sequencing BAC clones of salmon (*Salmo salar*) using the GS FLX (~250 bp read length), however, the potential of the method to result in high-quality BAC clone sequences was put in question [25].

Based on these initial studies the 454/Roche platform can be considered a robust platform to assemble genes from genomic sequences given sufficient sequence coverage. However, at sequencing capacity of up to 500 Mb per single GS FLX Titanium run the sequencing of individual BAC clones would be a rather non-economical approach and efficient use of the technology would require the possibility of multiplexing individual samples. Recently, pools of 28 BAC clones of wild rice *Oryza barthii*, selected from fingerprinted contigs, were sequenced by the 454 technology and assembled to superscaffolds by mapping to the *O. sativa *rice reference genome [26]. Due to the lack of a reference genome this BAC pool sequencing approach is not yet feasible for barley and multiplex sequencing would require a reliable tagging (barcoding) strategy to reveal sequence read and BAC clone origin relationships. Barcodes are specific short sequence tags that can be introduced either before the 454 sequencing library preparation [27] or by ligation of individual adaptors ("MID" = Multiplex Identifier", Roche Diagnostics) to fragmented BAC DNAs prior to sequencing in pools.

Here, as a proof of concept for a new strategic component of sequencing a large complex and highly repetitive crop plant genome in a clone-by-clone approach, we report the pool sequencing of 91 barcoded, randomly selected, gene containing barley BACs by the 454 technology. Furthermore, we present the assembly of the sequence data under variable parameters and evaluate the resulting assemblies for their consistency and reliability.

## Results

### Sequencing and preassembly processing

Initially, 91 non-overlapping BACs were selected based on the information to carry at least one gene [28]. Five of these clones, were previously Sanger-sequenced by others and deposited at NCBI Genbank (Additional file 1: Table S1). Whereas the sequence of one BAC clone (318G23) is an unfinished HTGS1 phase entry [29] encompassing 19 unordered contigs, the four others (184G09, 259I16, 631P08 and 711N16) were available as finished sequence and served as reference to monitor the quality of the 454 sequencing and assembly. In the following these four clones are referred to as 'reference BACs'.

In two different laboratories, one set each of 43 independent BACs plus the same five reference BACs were barcode tagged by two different strategies and sequenced in pools on Roche GS FLX sequencers.

**Figure 1:**
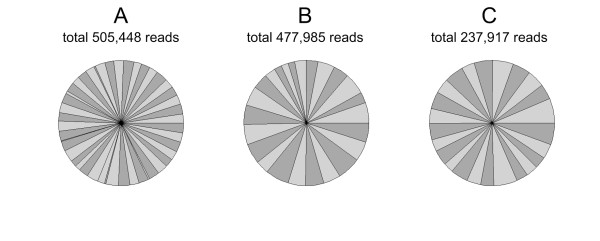
**Distribution of barcoded 454 sequencing reads per BAC**. (A) Set 1: two 24-BAC-pools on a full Roche 70 × 75 picotiterplate; (B) Set 2: two 12-BAC-pools on a half Roche 70 × 75 picotiterplate each; (C) Set 2: one 24-BAC-pool on a half Roche 70 × 75 picotiterplate.

For the vast majority of all reads (98.8% set 1; 94% set 2), the correct barcoding adaptor motifs could be recognized allowing the unambiguous assignment of sequences to the corresponding BACs. This resulted in 11,990 reads per clone on average except for 8 BACs of set 1 with less than 3,000 sequences per clone (Additional file 1: Table S1), prompting to re-sequence them. After that, altogether the BACs of both sets were represented by 1,221,350 reads (Table 1). Since absolute accuracy and lack of any bias in clone size estimation, barcoding, determination of DNA concentration, equimolar pooling and library preparation is rather impossible, deviations from the expected equal read recovery rate per BAC clone were expected. In fact, with a ratio of minimum to maximum number of reads per BAC of 1:16 (1,817/29,180 reads) we observed such deviations as illustrated in Fig. 1 and Additional file 1: Table S1.

**Table 1:** Summary of 454 sequencing for 96 BACs in two sets.

set	BAC	num reads	vec	coli	num reads w/o vec/coli	avgread length (bp)	min cov	max cov	av cov
1	48	505,448	3%	6%	460,168	225	12.5×	37.3×	21.6×
2	48	715,902	3%	10%	626,155	217	9.4×	64.4×	26.6×

both	96	1,221,350	3%	8%	1,086,323	221	9.4×	64.4×	24.3×

**Figure 2:**
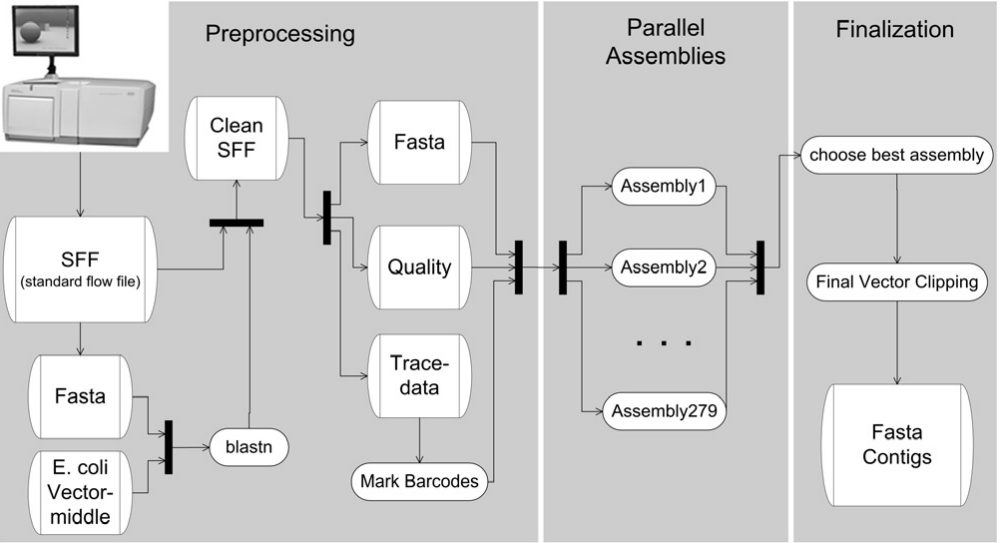
**Pipeline for assemblies of barcoded 454 BAC sequences**. Trace data, fasta sequences and sequence quality values are extracted from the raw data (sff). Vector and E. coli derived sequences and barcodes are marked. After preprocessing 279 assemblies with different parameter configurations are performed. Only the assembly with best results is kept for post-processing.

After clipping of the BAC specific barcodes the obtained sequences were processed following the assembly pipeline depicted in Fig. 2. In the preprocessing phase, reads derived from BAC vector and *E. coli *DNA were discarded (mean 3% and 8% of all reads, respectively) leaving 1,086,323 sequences with average read length of 221 bp (Table 1) for assembly. Presuming that the combined length of all contigs per assembly represented the overall BAC insert length, the obtained sequencing coverage by 454 reads ranged between 9.4× and 64.4× per individual BAC clone (ratio 1:7) with an average of 24.3×. If considering the accumulated contig length per BAC after assembly equals true insert length (see below and Additional file 1: Table S1) this coverage corresponded to average 10,610 reads per 100 kb insert.

### Mapping 454 sequences to Sanger references

To evaluate how completely and evenly the clone inserts were represented by the obtained sequences we mapped the 454 reads of the reference BACs from both sets to the complete Sanger sequences by BlastN [30] (Additional file 2: Table S2). Considering all BlastN matches with e-values < 10^-10^, altogether 908,757 out of 915,896 reference positions (99.985%) were hit by 454 reads. A similar result was obtained by evaluating exclusively the best BlastN alignments. If two or more hits with the same maximum match score were obtained, one of the alternative hits was randomly chosen. By this algorithm 915,705 reference positions (99.979%) were covered.

**Figure 3:**
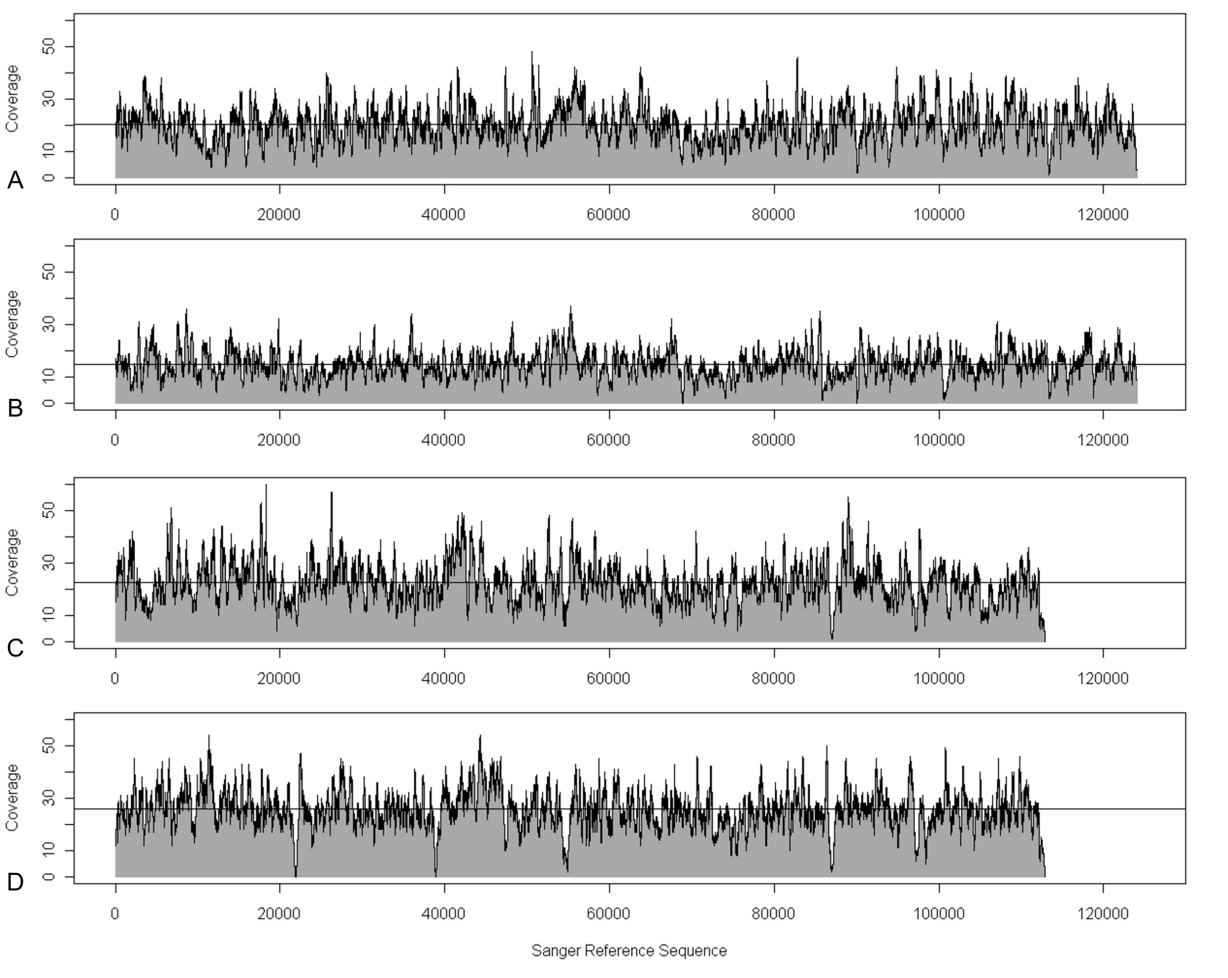
**Read distribution on Sanger Reference Sequences**. Reads for BAC 259I16 from set 1 (A), set 2 (B) and BAC 711N16 from set 1 (C) and set 2 (D) are mapped versus the Sanger reference sequence (Acc. no. AY268139, AF427791 (pos. 1 to 112,920)). The horizontal line indicates the average read coverage.

Examination of the BlastN alignments also provided distribution patterns of the 454 reads along the Sanger reference sequences, as exemplarily illustrated for BACs 259I16 and 711N16 from both sets (Fig. 3). For all eight alignments, 911,957 positions of the reference sequences (99.6%) were covered by at least five sequences. Only 3,939 positions (0.4%) were covered less than fivefold.

### Optimal assembly parameters differ both between BACs and sequence sets

**Figure 4:**
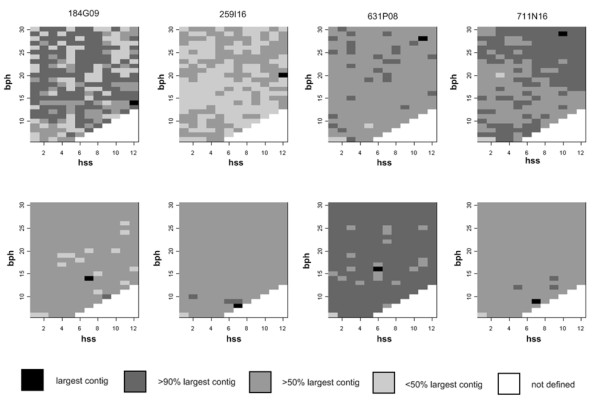
**Effect of different parameter settings on assemblies**. Heat maps visualizing the assembly results of 454 sequences of the four complete reference BACs (top: set 1; bottom: set 2) by MIRA under different combinations of hss (hash saving steps, X-axis) and bph (bases per hash, Y-axis). BACs from left to right are: 184G09, 259I16, 631P08, 711N16. Black fields indicate the hss/bph combinations resulting in the largest contig for the respective BAC, dark to light gray fields mark values producing a contig with >90%, >50% and <50% of the largest contig length, respectively. White fields represent meaningless combinations (hss > bph).

For assembly of the 454 sequences we tested initially the program Newbler (Roche), and subsequently MIRA [31]. Since all assemblies obtained with the latter program resulted in significantly higher N50/N80 contig lengths and less gaps when compared to the Sanger references, sequence assembly by Newbler was skipped for further analysis of the data. Using MIRA, hss and bph ("hash saving step" and "bases per hash", see methods section for details) were the two parameters which most strongly influenced contig numbers and lengths during assembly. However, while systematically changing the values of these parameters during assembly of all BAC clones, we were not able to identify a distinct or at least a restricted number of hss/bph combinations that outperformed others in regard to yielding the largest contigs for each individual BAC. The most suitable hss/bph combination to obtain the longest contigs differed between BACs as well as the set in which the data were generated (Fig. 4, Additional file 3: Figure S1, Additional file 4: Figure S2, Additional file 5: Figure S3).

### Sequence assembly under iterative parameter changes

**Figure 5:**
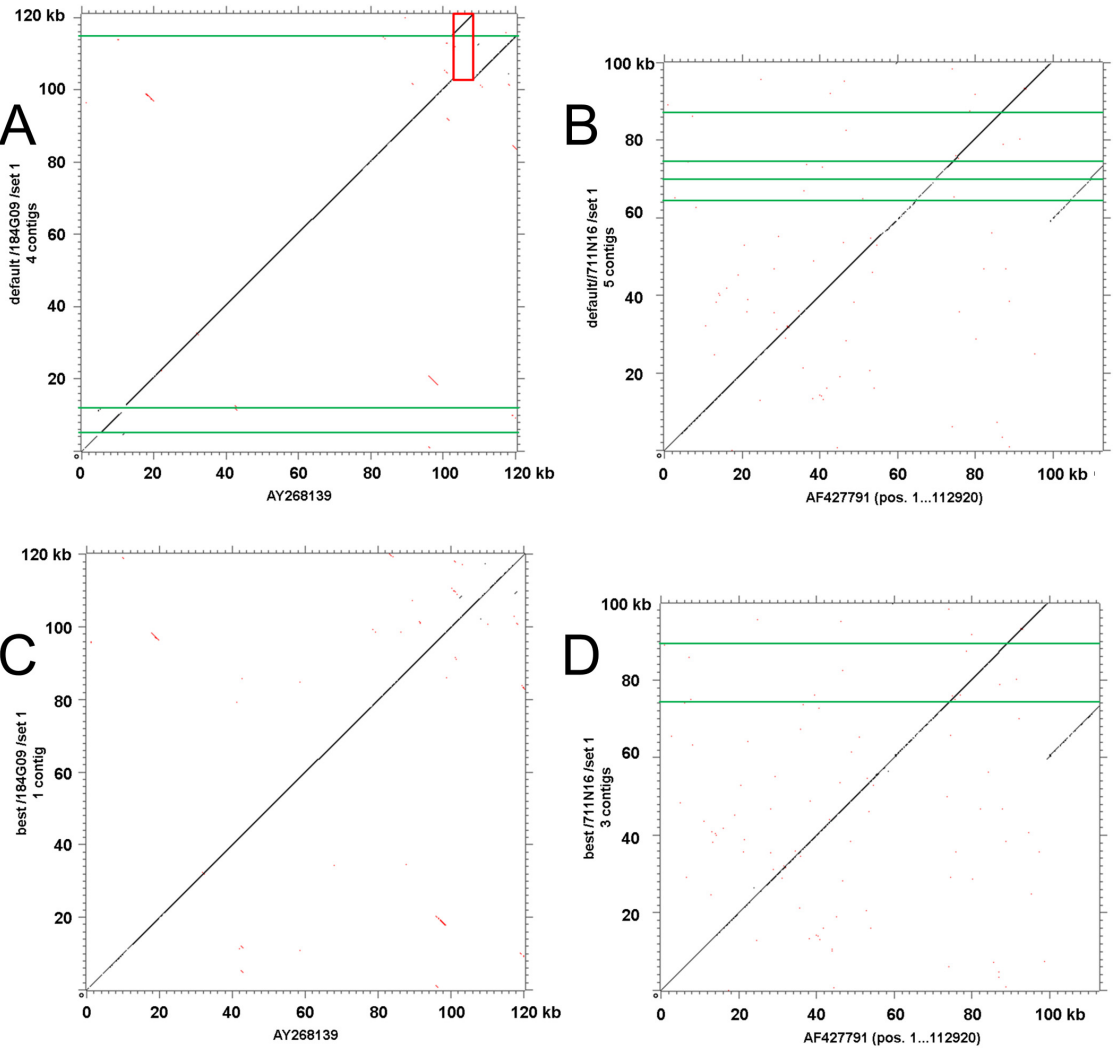
**Comparison of assembled contigs (y-axis) with the Sanger reference sequence (x-axis)**. Tuple plots for BACs 184G09 (A, C) and 711N16 (B, D). A and B: Mira assemblies with default parameters (hss 4, bph 16). C and D: Best MIRA assemblies after iterative parameter changes. Contigs larger than 1 kb were concatenated after directing and ordering by comparison to the Sanger reference sequence, horizontal green lines mark contig borders. Whereas in the default assembly of 184G09 (A) the longest contig is wrongly assembled in the region homolog to 102.5-108.0 kb (red rectangle) of the reference, the best assembly (C) results in a single contig accurately fitting the Sanger sequence. In contrast, the assembly problem in repetitive regions of 711N16 (B, D) even can not be resolved by the best assembly although turning out in less contigs ("collapsed" sequences, see text).

Since optimal *ab initio *assembly parameters could not be defined, the clipped sequences were assembled by MIRA with 279 iterative changes of the hss/bph parameter set for each of the 96 BAC data sets, resulting in 26,784 assemblies. For each clone, the assembly with the largest contig was defined as the 'best assembly' and selected for further analyses. As expected from the parameter optimization attempts (Fig. 4), this approach led to assemblies with less and larger contigs as compared with assemblies obtained under default settings (hss 4, bph 16) (Fig. 5, Additional file 6: Figure S4, Additional file 7: Figure S5).

**Figure 6:**
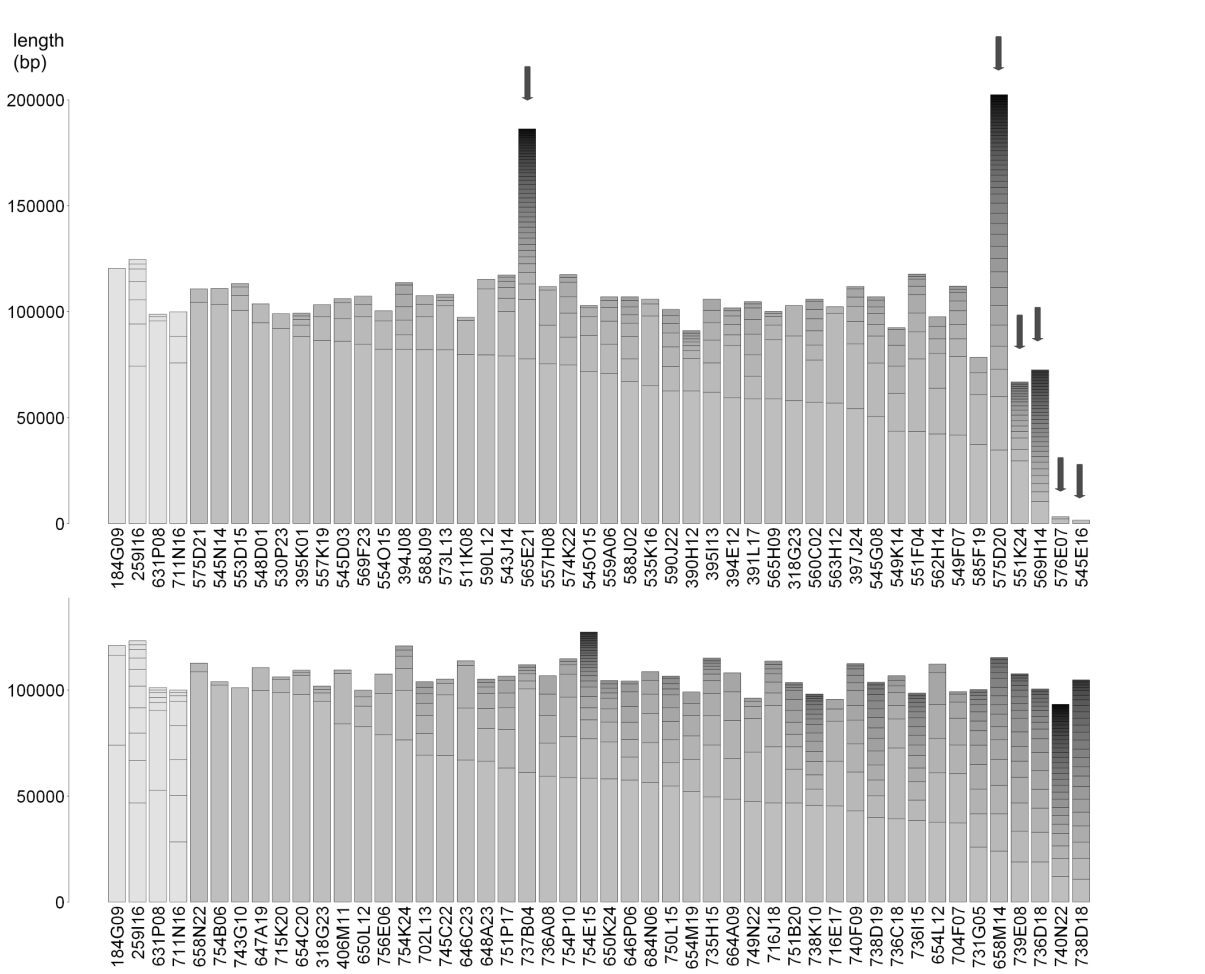
**Contig lengths per BAC**. Cumulative contig lengths per BAC for the 96 best MIRA assemblies after iterative parameter changes, shown for set 1 (upper panel) and set 2 (lower panel). Each bar represents one clone assembly and depicts all contigs >1,000 bp, ordered by ascending length from top to bottom and separated by horizontal dashes. The four Sanger sequenced reference BACs are shown most leftwards in both charts (from left to right 184G09, 259I16, 631P08, 711N16), arrows in the top chart indicate the six assembly outliers (see text).

For 68% (65 of 96) of the best assemblies, the largest contig spanned more than 50 kb. In 76 assemblies (79%), the number of contigs >1 kb was lower than 10 at a 21× mean sequence coverage, in 92 assemblies (96%) lower than 30 with a 20× mean coverage. Fig. 6 summarizes the 96 best assemblies by depicting all contigs larger than 1 kb.

Altogether, the 96 best alignments spanned 10,106,812 bp in 766 contigs >1 kb (Table 2) with N50>48.9 kb (N80 30.9 kb, N90 20.7 kb) and an average coverage of 22×. In terms of sequence quality 94%, 97% and 99% of the sequence were represented with Phred confidence values [32] above Q40, Q30 and Q20, respectively. A detailed inspection for sequencing errors was done by comparing the contigs of the 8 best MIRA assemblies with the Sanger reference sequences (Additional file 8: Table S3). Over a total length of 879,457 bp inspected sequence, 170 differences were found, thereof 39 single base exchanges and 131 single base insertions/deletions. This corresponded to a mean Phred confidence value of Q37, ranging from Q36 to Q40 among the 8 assemblies. The majority of sequencing errors was identified within homo-nucleotide stretches (112 differences, 66% of all). This type of error is known as intrinsic for the pyrosequencing based 454 technology. These errors could be resolved by additional sequences generated by other technologies.

**Table 2:** Summary of best MIRA assembly results for 96 BACs in two sets and four reference BACs included in both sets.

set	BAC	largest contig bp	sum of contigs >1 kb bp	N50 for contigs >1 kb bp	N80 for contigs >1 kb bp	N90 for contigs >1 kb bp	avg cov	0.5-10 kb	10-50 kb	50-100 kb	>100 kb
1	all (48)	66,682	4,972,546	59,359	34,037	21,204	22×	488	61	34	4
2	all (48)	58,210	5,134,266	38,959	27,816	20,140	27×	546	97	24	3

both	all (96)	62,466	10,106,812	48,945	30,926	20,671	24×	1.034	158	58	7

1	184G09	120,316	120,316	120,316	120,316	120,316	18×	-	-	-	1
1	259I16	74,161	125,700	74,161	11,492	8,588	19×	4	2	1	-
1	631P08	95,649	100,966	95,649	95,649	95,649	22×	2	-	1	-
1	711N16	75,684	101,682	75,684	12,600	11,564	24×	-	2	1	-

2	184G09	91,195	121,589	91,195	28,663	28,663	25×	1	1	1	-
2	259I16	44,395	131,950	10,249	7,210	5246	14×	9	4	-	-
2	631P08	52,664	102,141	26,440	18,577	18,577	24×	3	2	1	-
2	711N16	28,312	115,582	15,998	15,998	11,527	31×	4	5	-	-

The assemblies of six BACs from set 1 resulted in exceptionally high contig numbers or particularly short contig lengths compared to the other clones (Fig. 6 arrows and Additional file 9: Table S4). For two BACs (545E16, 576E07), this is due to low amounts of 454 reads even after re-sequencing (less than 2,500 per clone). Another two clones (565E21, 575D20) have considerably longer inserts than the others (>180 kb compared to ~110 kb as proved by restriction fingerprinting analysis) and were therefore not sufficiently covered by 454 reads to get satisfactory assemblies. For 551K24 a fair number of reads was obtained but the assembly represented only 87% of the insert length expected by restriction fingerprinting. Here, repetitive regions were assumed as underlying reason since parts of three contigs in the assembly are covered by more sequences than the two-fold median of the entire clone (Additional file 10: Figure S6). In the 569H14 assembly, parts of nearly all contigs were overrepresented by 454 reads indicating a high amount of repetitive structures hampering an assembly with less contigs (Additional file 11: Figure S7).

### Consistency of assemblies

To evaluate the assembly completeness and consistency, we compared in detail the best MIRA assemblies of the four reference BACs from both sets to their Sanger-derived sequences which were assumed to reflect the genuine BAC inserts. These comparisons resulted in eight tuple plots (Additional file 6: Figure S4) and showed that globally all parts of the reference sequence were represented in the 454 assemblies. Within the entire set of 45 contigs >1 kb we observed nine mis-assemblies in nine contigs (set 1: four, set 2: five). Four out of the five sequence parts affected by wrong assemblies in set 2 were identical to those in set 1 indicating that always the same repetitive structures were the underlying cause. Indeed, by closer inspection of the mis-assembled regions, different types of repetitive elements were identified which caused all incorrect assemblies (Fig. 5, other data not shown). Thus, by the chosen assembly strategy we can estimate that about 1.1 mis-assemblies (9 in 8 BAC assemblies) can be expected per BAC, preferentially in repetitive regions. Based on 9 mis-assembled contigs out of 45 in the reference BAC clones, about 153 out of 766 contigs >1 kb (20%) might be affected within the entire dataset of >10 Mb.

### Gene content and representation

To test to which degree the 87 *de novo *sequenced BAC clones contained genes and to measure the completeness and sequence integrity of the respective genes, we compared all contigs of the best MIRA assemblies with three protein (*Sorghum bicolor, Brachypodium distachyon, Oryza sativa*) [19,33,21] and two EST libraries (*Hordeum vulgare, Triticum aestivum*) [34]. The protein and EST references were aligned against the contigs using GenomeThreader [35] and the coverage of the query sequences against the homologous regions was measured as a proxy for completeness of the respective gene locus and to evaluate assembly correctness on gene containing regions on the *Hordeum *contigs (Additional file 12: Table S5).

On the contig level, *Brachypodium *proteins hit 109 contigs, rice 97 and *Sorghum *95 contigs. 115 contigs were hit by at least one protein entry, 84 were hit by all three libraries. On the BAC level, *Brachypodium *proteins hit 71 BACs, rice 69 and *Sorghum *66 BACs. A total of 73 out of 87 BACs hit at least one protein, 63 BACs contained at least one gene that showed similarity to all three databases. The reproducibly larger number of sequence matches with genes of *Brachypodium *was consistent with the closer phylogenetic relationship to barley compared to the two other fully sequenced grass reference genomes of rice and Sorghum [36]. A comparison to barley and wheat ESTs revealed 1,299 hits (1,154 unique) and 1,874 hits (1,558 unique), respectively.

**Figure 7:**
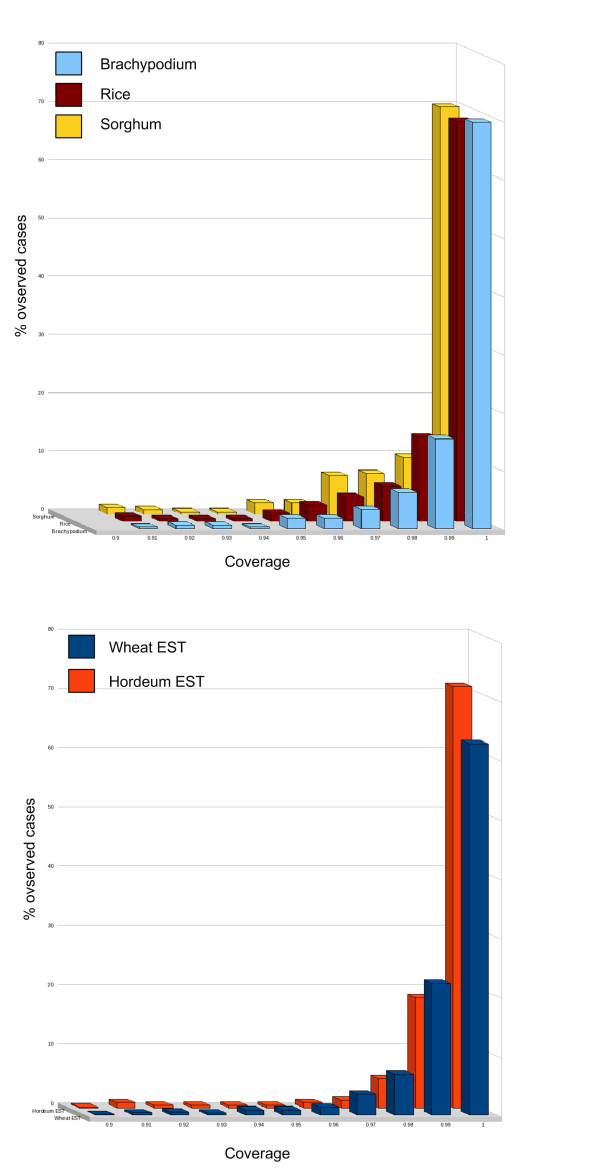
**Completeness of genes**. Coverage (completeness) of barley genes by comparison of best MIRA assembly contigs to protein orthologs of (A) *Brachypodium*, *Sorghum*, rice and (B) to ESTs of wheat and barley using GenomeThreader (28).

In total, 115 genes or gene fragments were identified. 84 (73%) were supported by hits to all grass model species (*Brachypodium*, rice and *Sorghum*), 18 (16%) to two and 13 (11%) to only one grass species. For 89 genes (77%), on average >0.95 of the gene was covered, for 112 genes (97%) ≥ 0.8. This indicated that the genomic sequences of the orthologous barley genes were almost completely represented on the contigs (Fig. 7). However, for three genes (2.6% of all) a representation <0.8 was determined, indicating a partial presence in the respective assemblies. To analyze the underlying reasons, the assemblies were manually inspected. In one gene locus a frameshift was found causing an interrupted alignment. Adjustment of the frameshift resulted in an uninterrupted reading frame with full gene coverage. The second case of under-representation has potentially been caused by a truncated pseudogene, as indicated by the absence of start and stop codon and the lack of complementary gene fragments on other contigs. Finally, for the third gene we found two complementary fragments located on two contigs from the respective BAC. Thus in summary, for 114 of 115 predicted genes (99%) the assembly resulted in coherent gene loci on individual contigs and only one gene appeared to be split between two contigs.

We also tested for consistency of exon order and orientation to identify potential mis-assemblies. No such events were observed. Thus, using gene containing regions as a measure we found no evidence for wrong assemblies indicated by missing exons, wrong exon order or orientation (flipped/reverse exons).

## Discussion

The high-throughput capacity of next generation sequencing (NGS) platforms promises new possibilities for sequencing large and complex genomes. Due to shorter read length as typically obtained in comparison to classical Sanger sequencing the full potential for *de novo *sequencing of plant genomes carrying more than 80% repetitive DNA, however, still needs to be demonstrated for NGS technologies. In the present pilot study we prove that 454 sequencing of pools of barcoded barley BACs and automated, sequence assembly can efficiently contribute to map-based clone-by-clone whole genome sequencing of a Triticeae genome. Sequencing of 91 barley BAC clones including four clones sequenced twice with independent strategies and comparison to the complete Sanger reference sequences revealed that 20-25 fold shotgun sequencing routinely lead to HTGS phase 1 assemblies (unfinished sequence containing gaps, order and relative orientation of the pieces not known; [29]). Furthermore, mapping of sequence reads to the Sanger references demonstrated the evenness of read distribution except for repetitive regions of high nucleotide identities.

### Sequence output and assembly conditions

Sequence read length is known to be one of the key parameters influencing the efficiency to sequence and assemble stretches of repetitive DNA of a genome. In the present study, reads with average lengths between 200-250 bp were obtained, which are typical for the 454/Roche GS FLX platform. At a 20-25-fold sequence coverage, the majority of the assemblies achieved HTGS phase I quality with N50 ~50 kb and less than 10 unordered contigs. This is comparable to a typical Sanger shotgun sequencing to 6-fold or higher coverage. Although it can not be ruled out that pre-selection of the clones for gene content may have positively influenced the assembly performance and results, this indicated that neither the moderate sequence lengths nor the high repeat content of the genome caused principle limitations for *de novo *sequencing of gene-containing barley BACs by the 454 technology.

Our results differed from those of another study describing the 454 sequencing of pooled but not barcoded salmon BAC clones [25]. Here, the obtained moderate contig lengths (N50 ~11.5 kb; largest contig ~34 kb) were interpreted as indication that considering 454 FLX sequencing without additional paired end sequences alone would provide a too limited strategy in context of a complex eukaryotic genome sequencing project. Yet to our understanding the moderate contig lengths could be caused by the limitations of the Newbler assembly software. In our study, with the sequences of the reference BACs, Newbler produced assemblies in which different copies of repetitive elements were not correctly separated. In contrast, MIRA [31] could resolve these structures and superior assemblies were obtained. However, not only the assembly algorithm influences contig length. Assembly quality is usually negatively correlated to the concentration of related repeated DNA elements in the target sequence. Pooling BAC clones without barcoding would extend the overall sequence length to be assembled and thus increases the chance for coincidental presence of members of highly conserved repetitive element families in the selected set of pooled BACs. This ultimately interferes with assembly quality and thus may explain to some extent the shorter assemblies obtained in case of Salmon/Newbler.

Applying iterative changes of different MIRA assembly parameters revealed that mainly hss (hash saving step) and bph (bases per hash) affected the outcome of the assemblies. Aiming for those with the largest contig per individual clone as the postulated best assemblies, the key parameters were found to be variable and unpredictable. This holds true not only for the assembly of different BACs but also when assembling the sequences of the same BAC from different sequencing sources (sets 1 and 2). Different hss and bph parameters leading to the best assemblies of different BACs indicated that at a given minimum sequence coverage individual features like repeat content and/or composition may essentially influence the quality of the assembly. The fact that different sequence sets from the same BAC - although both being representative for the sequenced clone - required different parameters for yielding a 'best' assembly, suggested that experimental and stochastic conditions like fragmentation profile, library representativeness and sequence length distribution influenced the outcome of the assemblies. Furthermore, it cannot be ruled out that the sequential order of individual reads fed into the assembly controlled the final outcome of the assembly.

With respect to these findings, iterative assembly with as many as reasonable parameter permutations as carried out in our study, seemed appropriate while consuming considerable computing time and performance. Selection of the best assembly as the one with the largest contig might be questionable, since production of chimeric contigs can be suspected and other metrics are probably better indicators for a low number of mis-assemblies. To address this we counted mis-assemblies and chimeric contigs in assemblies selected by the best N50, N80, and N90 values and by default parameters, respectively (Additional file 13: Table S6). As a result, compared to the largest contig metric (nine mis-assemblies in our reference BACs) N50, N80 and N90 led to similar numbers (nine, eight and nine, respectively) and application of the default MIRA parameters even produced 11 errors. Furthermore, except for one case (184G09 set2), none of the alternative metrics generated less chimeric contigs than the respective largest contig assemblies. This indicated that selection of the latter metrics did not warrant for the lowest number of mis-assemblies but was indeed a reasonable criterion due to lack of expensive validation experiments.

### Assemblies, genes and repeat structures

For *de novo *sequenced BACs we demonstrated that all genes present on the clones were practically completely represented in the best MIRA assemblies. Furthermore, with the exception of one gene, all ORFs were found uninterrupted by gaps between contigs. This outcome has been postulated before in another pilot study applying the Roche/454 GS20 sequencing to barley BACs [24]. Therefore, the selected approach of pooled BAC sequencing should enable to survey the entire gene content of the targeted BACs. Other, maybe more cost effective, approaches of reduced representation sequencing were published that are suited to survey gene content of a complex genome (for review see [19]). However, our presented strategy of barcoded BAC-pool sequencing delivered not only fully assembled gene sequences instead of partial gene information but could also serve as the basal approach for whole genome clone-by-clone sequencing in complex plant genomes.

On the other hand, comparing the assemblies of the four reference BACs to the Sanger-derived sequences also revealed limitations of the automated assembly of complex repetitive DNA regions. Mis-assemblies in these clones were observed preferentially in LTR regions of retrotransposons. LTR transposons are very frequent in barley and the occurrence of such elements with nucleotide identities of up to >99% in several copies is known to complicate even the assembly of Sanger sequenced BACs. Wrong assemblies in regions harboring such elements typically can occur in two ways:

(I) Sequences derived from different copies of the repeat "collapse" into one single region, finally "shortening" the consensus sequence (shown for BAC 711N16 in Fig. 5B, D). Since this normally should be accompanied by significantly increased sequence coverage in the "collapsed" region, those effects are possibly amenable to automated pattern recognition and thus could become corrected by reassembly under modified conditions. The "collapse" assembly problem is a strong argument for the selection of the assembly producing the largest contig as the optimal one. However, the fact that the best assembly of 184G09 (set 2) produced the largest contig by falsely resolving the retrotransposon structure (Additional file 6: Figure S4 b), whereas, in contrast, the default assembly produced shorter contigs but no mis-assembly (Additional file 7: Figure S5 j), indicated that the "largest-is-best-rule" remains a simplification and is not universally valid.

(II) Repetitive sequences are correctly split into different copies but the copies are arranged in the wrong order and/or direction. This type of incorrect assembly can not be detected without the availability of a reference sequence or knowledge about the involved types of repeats.

In both cases the use of paired end/mated pair sequencing approaches may help to overcome such shortcomings in the future. Paired-end sequencing applications are available and protocols for the preparation of libraries with read distances of ~200 bp ("paired end") and 2-5 kb ("mated pairs") were successfully applied [37-39]. In combination with the barcoding step this strategy may introduce inacceptable additional labour to the BAC pool sequencing. Therefore, additional non-barcoded paired end/mate-pair libraries could be prepared for an alternative higher throughput, shorter read NGS platform from the same sets of BACs which were already processed as barcoded pools by the 454 technology. Assembly errors in structural variants could be resolved in a similar context by deep coverage paired end Illumina sequencing with different fragment size in human whole genome sequencing [12]. In our case, as an example, the untagged fragments of 2 pools of 48 BACs (~10.6 Mb non redundant sequence) could be mixed and converted into Illumina paired end and/or mate pair libraries, respectively. Sequencing of such a library in just one lane on the Illumina GAII platform usually could produce 2 × 8 Mio. reads at affordable costs. This would deliver clone coverages between ~150× for a 200 bp distance paired end library and ~1,500× for a 2 kb distance mate pair library. Reads from the 200 bp insert library may only resolve errors in homopolymer stretches known as a methodological shortcoming of the 454 pyrosequencing method. In contrast, sequences from the longer distance mate pair library will help to bridge and order contigs of the 454 assemblies since the distance between the corresponding reads should be larger than repetitive elements hampering the joining of contigs. Furthermore, assembly errors will be identified by verification of direction and distance of the mate read pairs. Such a mixed two step strategy should allow the establishment of a largely automated BAC assembly and verification pipeline.

## Conclusion

Proving the initial concept, our study has demonstrated that multiplex sequencing of barcoded BACs by the 454 technology is appropriate for a clone based strategy of sequencing complex plant genomes. Though the content of complex repeat structures causes pitfalls for the assembly process, the sequence information obtained with a >20× coverage was highly representative for gene containing barley BACs by producing assemblies with N50 ~50 kb (N80 ~31 kb, N90 ~21 kb) and Q40 ~94%, which in most cases yielded phase I assemblies (unordered contigs, less than 10 gaps). Whereas the majority of contigs were expected to be correctly assembled in their gene loci, BACs harbouring complex repetitive structures will require additional experimental efforts to yield final assemblies. This may encompass paired end genomic sequencing approaches as well as gap closure by primer walking and Sanger sequencing of individual reads.

## Methods

### BAC sequencing

91 BACs derived from a barley BAC library [40] were sequenced in two independent sets of 48 BACs (Additional file 1: Table S1) containing the same five BAC clones for which reference sequences were generated by the Sanger method and are available at NCBI Genbank (Additional file 1: Table S1; BACs 1-5). Set 1 (BACs 1-48) was sequenced in two pools by proprietary protocols at Eurofins MWG Biotech [41] whereas set 2 (BACs 1-5 and 49-91) was sequenced following the procedure described below. Sequence assembly was performed for all clones under the same procedures as described below. The sequences of all 91 BACs were submitted to NCBI Genbank as raw data as well as and assembled unordered contigs larger than 0.5 kb (NCBI short read archive ID: SRP001149; Genome Project 37963).

#### BAC preparation

DNAs of the clones reported in this study (BACs 1-5 and 49-91) were prepared by an adapted "Maxi-Prep" protocol which after sequencing resulted in *E. coli *read ratios of 8% on average (Additional file 1: Table S1). Although not atypical for BAC preparations and suitable for the high throughput sequencing process, the procedure was optimized and applied to more than 1,000 barley BACs. Sequencing of these BACs resulted in a much lower mean *E. coli *sequence ratio of 3.1% with only 26 out of 1,028 clones (2.5%) contaminated by >10% of *E. coli *reads (data not shown). The following protocol describes this optimized version: The BACs were incubated in 2 × 20 ml TB (6 μg/mL chloramphenicol) for 15 hours at 325 rpm (36°C) and centrifuged for 10 min at 4000 rpm. The pellets were completely re-suspended on ice in 4 mL 10 mM EDTA. 8 mL 1% SDS/0.2 M NaOH were added without mixing and left on ice for 10 min followed by addition of 6 mL 3 M potassium acetate (15 min on ice). After centrifugation for 20 min at 4000 rpm the supernatant was filtered, 12 mL isopropanol were added and the solution was centrifuged again for 15 min at 4000 rpm. Re-suspension of the pellet was done in 1.5 mL 10 mM Tris/50 mM EDTA and 1 mL 7.5 M potassium acetate and the mixture was frozen for 30 min at -80°C. After re-thawing the suspensions were centrifuged for 15 min at 4000 rpm. The supernatant was transferred to 5 mL 96% ethanol, centrifuged for 10 min at 3500 rpm, and the pellet was re-suspended in 400 μl 50 mM Tris/50 mM EDTA. After incubation with 50 μg RNAse for 1 h at 37°C, threefold extraction with phenol/chloroforme/isoamyl alcohol (400 μl each; centrifugation 10 min 14000 rpm) was carried out. 280 μl isopropanol were added and the tube was kept at -20°C over night. After centrifugation (10 min 14000 rpm) and washing with 500 μl 80% ethanol the pellet was air-dried and dissolved in 200 μl TE by shaking 1 h at 42°C. The second precipitation was performed with 20 μL 1 M NaCl/550 μL 96% ethanol for 20 min at -80°C, followed by centrifugation (10 min 14000 rpm) and washing (500 μL 80% ethanol). After air-drying the pellet was dissolved in 50 μl TE.

Insert sizes of BAC clones were determined by pulsed-field agarose gel-electrophoresis (PFGE) in 1% UltraPure™-agarose (Invitrogen), 18 h, 120°, 5/15 sec switchtimes, 6 V/cm and 14°C. NotI restriction was performed with 200 ng of BAC-plasmid-DNA, 5 Units of *Not*I (New England Biolabs), 1× NEBuffer 3, 1 μg/ml BSA for 4 h.

#### Barcoding of BAC DNA fragments

About 5 μg of each BAC DNA were individually fragmented for 1 min at 3 bar N_2 _by nebulizers (part of GS DNA Library Preparation Kit, Roche Diagnostics) resulting in average fragment lengths of 700-800 bp. The nebulized DNAs were purified by MinElute columns (Qiagen) and eluted in 20 μl TE each. Blunt end repair, ligation of the barcoding adaptors to the fragments as well as all other steps prior preparation of the 454 sequencing library were carried out essentially as described in [27] using 500 ng of each nebulized DNA as starting material (Quant-IT^® ^PicoGreen^® ^ds DNA assay; Invitrogen). Barcode oligonucleotides (desalted) were purchased at Metabion (Martinsried, Germany) and consisted of the barcode (3'end, Additional file 14: Table S7) and its reverse complement (5'end), separated by the *Srf*I recognition site 5'-GCCCGGGC-3'. After careful quantification of the barcoded BAC-DNAs by the PicoGreen^® ^assay, 10 ng of each BAC-DNA were pooled and the pool was proceeded to the dephosphorylation step, followed by *Srf*I digest and small fragment removal.

#### 454 sequencing of barcoded BAC fragment pools

The SrfI digested and purified BAC fragment pool DNA was used to prepare the 454 sequencing library using the GS DNA Library Preparation Kit, following the instructions of the GS FLX Shotgun DNA Library Preparation Manual (Roche Diagnostics). Single stranded 454 sequencing libraries were quantified by a qPCR assay [42] and processed by emulsion PCR and sequencing as described in the GS FLX manuals (Roche Diagnostics). The BACs of set 2 were sequenced in pools of 12 clones (BACs 49-60; BACs 61-72) and 24 clones (BACs 1-5 and 73-91, respectively, loading three times a half 70 × 75 Picotiterplate (PTP) with 900,000 beads each.

### Sequence processing and assemblies

As shown in Fig. 2, prior to assembly, all 454 reads were screened for DNA from vector and *E. coli *by BlastN [30] against the sequence of pBeloBAC11 (GenBank U51113, pos. 2391...5890, i.e. the part from 2 kb downstream to 2 kb upstream of the HindIII cloning site) and the genomes of *E. coli *DH10B (GenBank:NC_010473) and K-12 sub-strain MG1655 (GenBank:U00096). All 454 sequences with matches e-value < 10^-10 ^were excluded from the subsequent assemblies. Vector sequences derived from regions GenBank U51113, pos. 1...2390 and 5891...7507 were retained in the datasets for assembly by this approach in order to get complete representation of the vector-insert spanning clone regions. Contigs in the BAC assemblies containing these vector sequence parts were then shortened to the cloning site. In addition, the barcoding motifs as well as low quality regions identified by the GS FLX software were marked to be ignored for the assembly.

The pre-processed reads were assembled by Newbler (Roche), version 2.0, and MIRA, developmental version 2.9.26x3 [31]. Assemblies were run on a high performance 42 nodes Linux Cluster, consisting of 4 GB RAM Dual Opteron computers using Rocks4 as operating system. For MIRA, Skim3 Parameters were fitted individually for each BAC. Skim3 as part of the MIRA program is an algorithm related to SSAHA [43], which is used for a pre-selection of related reads. This is done by converting reads into hashes and storing them in a table. Length of each hash-word is given by the parameter "bases per hash" (bph) and the granularity of words being stored per read is given by "hash saving steps" (hss). Several tests showed that the quality of an assembly depends essentially on these parameters. Since best fitting parameters are hardly to predict, all combinations were executed. The parameter ranges were 6-30 for bph and 1-12 for hss. Given that using in combination a larger hss than bph value is not making any sense, we performed 279 assemblies for each BAC.

### Sequence comparisons

Mapping the 454 reads to the Sanger reference sequences were performed by BlastN [30] and the read distribution was visualized by R, a statistical computing and graphics language http://www.r-project.org/. Sequence homology plots were generated by tuple plot [44]. Alignments of the assembled sequences against protein and EST databases were performed using GenomeThreader [35]. Alignment coverage values were taken from GenomeThreader with values > 1.0 representing slightly larger exons in barley compared to the respective reference. Those values have been accumulated into the 1.0 value for graphical representation.

## Authors' contributions

NS conceived the project in collaboration with AG, KM, MP and US and supervised its progress. RA and DS were in charge for BAC fingerprinting and selection for sequencing. ST led the preparation of BAC DNA pools and 454 sequencing and carried out the pre-processing of the sequence raw data. BS, AP and US developed and applied the assembly pipeline. The data analyses were performed by BS, MF, HG, KM, MS and NS. The manuscript was written by BS, ST, KM, MP and NS. All authors read and approved the final manuscript.

## Supplementary Material

Additional file 1 **Table S1. Clone and sequencing read data**. Various information about Clones, raw data and assemblies. Click here for file

Additional file 2 **Table S2. BlastN of 454 reads against BAC reference sequences**. Raw 454 reads were mapped on Sanger reference sequences vis BlastN. The table number of covered and uncovered positions on the reference. Click here for file

Additional file 3 **Figure S1. Heat maps of N50 lengths of different assemblies**. Heat maps visualizing the assembly results of 454 sequences of the four complete reference BACs (top: set 1; bottom: set 2) by MIRA under different combinations of hss (hash saving steps, X-axis) and bph (bases per hash, Y-axis). BACs from left to right are: 184G09, 259I16, 631P08, 711N16. Black fields indicate the hss/bph combinations resulting in the highest N50 values for the respective BAC. Dark to light gray fields mark values producing a contig with >90%, >50% and <50% of these values, respectively. White fields represent meaningless combinations (hss > bph). Click here for file

Additional file 4 **Figure S2. Heat maps of N80 lengths of different assemblies**. Heat maps visualizing the assembly results of 454 sequences of the four complete reference BACs (top: set 1; bottom: set 2) by MIRA under different combinations of hss (hash saving steps, X-axis) and bph (bases per hash, Y-axis). BACs from left to right are: 184G09, 259I16, 631P08, 711N16. Black fields indicate the hss/bph combinations resulting in the highest N80 values for the respective BAC. Dark to light gray fields mark values producing a contig with >90%, >50% and <50% of these values, respectively. White fields represent meaningless combinations (hss > bph). Click here for file

Additional file 5 **Figure S3. Heat maps of N90 lengths of different assemblies**. Heat maps visualizing the assembly results of 454 sequences of the four complete reference BACs (top: set 1; bottom: set 2) by MIRA under different combinations of hss (hash saving steps, X-axis) and bph (bases per hash, Y-axis). BACs from left to right are: 184G09, 259I16, 631P08, 711N16. Black fields indicate the hss/bph combinations resulting in the highest N90 values for the respective BAC. Dark to light gray fields mark values producing a contig with >90%, >50% and <50% of these values, respectively. White fields represent meaningless combinations (hss > bph). Click here for file

Additional file 6 **Figure S4. Tupleplots of best MIRA assemblis versus Sanger reference sequence**. Tupleplots show comparisons of best MIRA assembly contigs > 1 kb (y-axis) to the complete Sanger reference sequences (x-axis). a) 184G09 Set1/AY268139; b) 184G09 Set2/AY268139; c) 259I16 Set1/AF474373; d) 259I16 Set2/AF474373; e) 631P08 Set1/DQ249273; f) 631P08 Set2/DQ249273; g) 711N16 Set1/AF427791 (pos. 1...112.920); h) 711N16 Set2/AF427791 (pos. 1...112.920). Click here for file

Additional file 7 **Figure S5. Tupleplots of default MIRA assemblis versus Sanger reference sequence**. Tupleplots show comparisons of best MIRA assembly contigs > 1 kb (y-axis) to the complete Sanger reference sequences (x-axis). i) 184G09 Set1/AY268139; j) 184G09 Set2/AY268139; k) 259I16 Set1/AF474373; l) 259I16 Set2/AF474373; m) 631P08 Set1/DQ249273; n) 631P08 Set2/DQ249273; o) 711N16 Set1/AF427791 (pos. 1...112.920); p) 711N16 Set2/AF427791 (pos. 1...112.920). Click here for file

Additional file 8 **Table S3. Sequencing errors in Mira assemblies**. Assessment of sequencing error rate by comparing contigs of the best MIRA assemblies with the Sanger reference sequences. Click here for file

Additional file 9 **Table S4. Assembly status of outliers within BAC set1 and insert sizes estimated by fingerprinting**. Simple statistics about the outliers in BAC set1 show reasons in four of six cases. In those cases low coverage led to a high number of contigs. The low coverage resulted from either a very low number of reads or a very large insert size of the BAC. Click here for file

Additional file 10 **Figure S6. Coverage Diagram of BAC 551K24**. Coverage of the best MIRA assembly contigs by 454 reads from BAC 551K24, set1. Black vertical lines separate the contigs; red horizontal lines indicate the median and the twofold median, respectively. Click here for file

Additional file 11 **Figure S5. Coverage Diagram of BAC 569H14**. Coverage of the best MIRA assembly contigs by 454 reads from BAC 569H14, set1. Black vertical lines separate the contigs; red horizontal lines indicate the median and the twofold median, respectively. Click here for file

Additional file 12 **Table S5. Coverages (completeness) of genes and gene parts calculated by comparison of best MIRA assembly contigs to the protein database of B. distachyon, O. sativa and S. bicolor using Genome Threader**. Click here for file

Additional file 13 **Table S6. Number of misassemblies/chimeric contigs using different MIRA assembly metrics**. Misassemblies and chimeric contigs were counted by visual inspection of tuple-plots against Sanger reference sequences. Largest contig, largest N50, N80 and N90 contigs and default parameter setting were regarded as possible metrics. Click here for file

Additional file 14 **Table S7. Barcodes for BACs of set2**. The table shows the list of barcodes that were used for tagging different BACs in set 2. Click here for file

## References

[B1] Food And Agriculture Organization Of The United Nations http://faostat.fao.org/

[B2] Schulte D Close TJ Graner A Langridge P Matsumoto T Muehlbauer G Sato K Schulman AH Waugh R Wise RP Stein N The international barley sequencing consortium - at the threshold of efficient access to the barley genome Plant Physiol 2009 149 142 147 10.1104/pp.108.128967 19126706 PMC2613708

[B3] Wicker T Taudien S Houben A Keller B Graner A Platzer M Stein N A whole-genome snapshot of 454 sequences exposes the composition of the barley genome and provides evidence for parallel evolution of genome size in wheat and barley Plant J 2009 59 712 722 10.1111/j.1365-313X.2009.03911.x 19453446

[B4] Sanger F Nicklen S Coulson AR DNA sequencing with chain-terminating inhibitors Proc Natl Acad Sci USA 1977 74 5463 5467 10.1073/pnas.74.12.5463 271968 PMC431765

[B5] Eversole K Graner A Stein N Feuillet C, Muehlbauer J Wheat and barley genome sequencing Genetics and genomics of the Triticeae 2009 7 Springer 713 742 full_text

[B6] Mardis ER Next-generation DNA sequencing methods Annu Rev Genomics Hum Genet 2008 9 387 402 10.1146/annurev.genom.9.081307.164359 18576944

[B7] Shendure J Ji H Next-generation DNA sequencing Nat Biotechnol 2008 26 1135 1145 10.1038/nbt1486 18846087

[B8] Ansorge WJ Next-generation DNA sequencing techniques N Biotechnol 2009 25 195 203 10.1016/j.nbt.2008.12.009 19429539

[B9] MacLean D Jones JDG Studholme DJ Application of 'next-generation' sequencing technologies to microbial genetics Nat Rev Microbiol 2009 7 287 296 10.1038/nrmicro2088 19287448

[B10] Ley TJ Mardis ER Ding L Fulton B McLellan MD Chen K Dooling D Dunford-Shore BH McGrath S Hickenbotham M Cook L Abbott R Larson DE Koboldt DC Pohl C Smith S Hawkins A Abbott S Locke D Hillier LW Miner T Fulton L Magrini V Wylie T Glasscock J Conyers J Sander N Shi X Osborne JR Minx P Gordon D Chinwalla A Zhao Y Ries RE Payton JE Westervelt P Tomasson MH Watson M Baty J Ivanovich J Heath S Shannon WD Nagarajan R Walter MJ Link DC Graubert TA DiPersio JF Wilson RK DNA sequencing of a cytogenetically normal acute myeloid leukaemia genome Nature 2008 456 66 72 10.1038/nature07485 18987736 PMC2603574

[B11] Wang J Wang W Li R Li Y Tian G Goodman L Fan W Zhang J Li J Zhang J Guo Y Feng B Li H Lu Y Fang X Liang H Du Z Li D Zhao Y Hu Y Yang Z Zheng H Hellmann I Inouye M Pool J Yi X Zhao J Duan J Zhou Y Qin J Ma L Li G Yang Z Zhang G Yang B Yu C Liang F Li W Li S Li D Ni P Ruan J Li Q Zhu H Liu D Lu Z Li N Guo G Zhang J Ye J Fang L Hao Q Chen Q Liang Y Su Y San A Ping C Yang S Chen F Li L Zhou K Zheng H Ren Y Yang L Gao Y Yang G Li Z Feng X Kristiansen K Wong GK-S Nielsen R Durbin R Bolund L Zhang X Li S Yang H Wang J The diploid genome sequence of an Asian individual Nature 2008 456 60 65 10.1038/nature07484 18987735 PMC2716080

[B12] Bentley DR Balasubramanian S Swerdlow HP Smith GP Milton J Brown CG Hall KP Evers DJ Barnes CL Bignell HR Accurate whole human genome sequencing using reversible terminator chemistry Nature 2008 456 53 59 10.1038/nature07517 18987734 PMC2581791

[B13] Wheeler DA Srinivasan M Egholm M Shen Y Chen L McGuire A He W Chen Y-J Makhijani V Roth GT Gomes X Tartaro K Niazi F Turcotte CL Irzyk GP Lupski JR Chinault C zhi Song X Liu Y Yuan Y Nazareth L Qin X Muzny DM Margulies M Weinstock GM Gibbs RA Rothberg JM The complete genome of an individual by massively parallel DNA sequencing Nature 2008 452 872 876 10.1038/nature06884 18421352

[B14] Ossowski S Schneeberger K Clark RM Lanz C Warthmann N Weigel D Sequencing of natural strains of Arabidopsis thaliana with short reads Genome Res 2008 18 2024 2033 10.1101/gr.080200.108 18818371 PMC2593571

[B15] Velasco R Zharkikh A Troggio M Cartwright DA Cestaro A Pruss D Pindo M Fitzgerald LM Vezzulli S Reid J Malacarne G Iliev D Coppola G Wardell B Micheletti D Macalma T Facci M Mitchell JT Perazzolli M Eldredge G Gatto P Oyzerski R Moretto M Gutin N Stefanini M Chen Y Segala C Davenport C Demattè L Mraz A Battilana J Stormo K Costa F Tao Q Si-Ammour A Harkins T Lackey A Perbost C Taillon B Stella A Solovyev V Fawcett JA Sterck L Vandepoele K Grando SM Toppo S Moser C Lanchbury J Bogden R Skolnick M Sgaramella V Bhatnagar SK Fontana P Gutin A de Peer YV Salamini F Viola R A high quality draft consensus sequence of the genome of a heterozygous grapevine variety PLoS One 2007 2 e1326 10.1371/journal.pone.0001326 18094749 PMC2147077

[B16] Wicker T Zimmermann W Perovic D Paterson AH Ganal M Graner A Stein N A detailed look at 7 million years of genome evolution in a 439 kb contiguous sequence at the barley Hv-eIF4E locus: recombination, rearrangements and repeats Plant J 2005 41 184 194 10.1111/j.1365-313X.2004.02285.x 15634196

[B17] Ma Y Sun H Zhao G Dai H Gao X Li H Zhang Z Isolation and characterization of genomic retrotransposon sequences from octoploid strawberry (Fragaria × ananassa Duch) Plant Cell Rep 2008 27 499 507 10.1007/s00299-007-0476-7 18026732

[B18] Strawberry Functional Genomics at Virginia Tech http://strawberry.vbi.vt.edu/tiki-index.php

[B19] Paterson AH Bowers JE Bruggmann R Dubchak I Grimwood J Gundlach H Haberer G Hellsten U Mitros T Poliakov A Schmutz J Spannagl M Tang H Wang X Wicker T Bharti AK Chapman J Feltus FA Gowik U Grigoriev IV Lyons E Maher CA Martis M Narechania A Otillar RP Penning BW Salamov AA Wang Y Zhang L Carpita NC Freeling M Gingle AR Hash CT Keller B Klein P Kresovich S McCann MC Ming R Peterson DG ur Rahman M Ware D Westhoff P Mayer KFX Messing J Rokhsar DS The Sorghum bicolor genome and the diversification of grasses Nature 2009 457 551 556 10.1038/nature07723 19189423

[B20] The Arabidopsis Genome Initiative Analysis of the genome sequence of the flowering plant Arabidopsis thaliana Nature 2000 408 796 815 10.1038/35048692 11130711

[B21] International Rice Genome Sequencing Project The map-based sequence of the rice genome Nature 2005 436 793 800 10.1038/nature03895 16100779

[B22] Maize Genetics and Genomics Database http://www.maizegdb.org

[B23] International Barley Sequencing Consortium http://barleygenome.org

[B24] Wicker T Schlagenhauf E Graner A Close TJ Keller B Stein N 454 sequencing put to the test using the complex genome of barley BMC Genomics 2006 7 275 10.1186/1471-2164-7-275 17067373 PMC1633745

[B25] Quinn N Levenkova N Chow W Bouffard P Boroevich K Knight J Jarvie T Lubieniecki K Desany B Koop B Harkins T Davidson W Assessing the feasibility of GS FLX Pyrosequencing for sequencing the Atlantic salmon genome BMC Genomics 2008 9 404 10.1186/1471-2164-9-404 18755037 PMC2532694

[B26] Rounsley S Marri PR Yu Y He R Sisneros N Goicoechea JL Lee SJ Angelova A Kudrna D Luo M Affourtit J Desany B Knight J Niazi F Egholm M Wing RA De novo next generation sequencing of plant genomes Rice 2009 2 35 43 10.1007/s12284-009-9025-z

[B27] Meyer M Stenzel U Hofreiter M Parallel tagged sequencing on the 454 platform Nat Protoc 2008 3 267 278 10.1038/nprot.2007.520 18274529

[B28] Madishetty K Condamine P Svensson JT Rodriguez E Close TJ An improved method to identify BAC clones using pooled overgos Nucleic Acids Res 2007 35 e5 10.1093/nar/gkl920 17151072 PMC1761434

[B29] NCBI High-Throughput Genomic Sequences http://www.ncbi.nlm.nih.gov/HTGS/

[B30] Altschul SF Gish W Miller W Myers EW Lipman DJ Basic local alignment search tool J Mol Biol 1990 215 403 410 2231712 10.1016/S0022-2836(05)80360-2

[B31] MIRA 2 - Whole Genome Shotgun and EST Sequence Assembler http://chevreux.org/projects_mira.html

[B32] Ewing B Green P Base-calling of automated sequencer traces using phred. II. Error probabilities Genome Res 1998 8 186 194 9521922

[B33] Brachipodium distachyon http://www.brachypodium.org/

[B34] Close TJ Wanamaker S Roose ML Lyon M HarvEST Methods Mol Biol 2007 406 161 177 full_text 18287692 10.1007/978-1-59745-535-0_7

[B35] Gremme G Brendel V Sparks ME Kurtz S Engineering a software tool for gene structure prediction in higher organisms Information and Software Technology 2005 47 965 978 10.1016/j.infsof.2005.09.005

[B36] Bolot S Abrouk M Masood-Quraishi U Stein N Messing J Feuillet C Salse J The 'inner circle' of the cereal genomes Curr Opin Plant Biol 2009 12 119 125 10.1016/j.pbi.2008.10.011 19095493

[B37] Illumina Paired-End Sequencing http://www.illumina.com/pages.ilmn?ID=329

[B38] Farrer RA Kemen E Jones JD Studholme DJ De novo assembly of the Pseudomonas syringae pv. syringae B728a genome using Illumina/Solexa short sequence reads FEMS Microbiol Lett 2009 291 103 11 10.1111/j.1574-6968.2008.01441.x 19077061

[B39] Shen Y Sarin S Liu Y Hobert O Pe'er I Comparing platforms for C. elegans mutant identification using high-throughput whole-genome sequencing PLoS One 2008 3 e4012 10.1371/journal.pone.0004012 19107202 PMC2603312

[B40] Yu Y Tomkins JP Waugh R Frisch DA Kudrna D Kleinhofs A Brueggeman RS Muehlbauer GJ Wise RP Wing RA A bacterial artificial chromosome library for barley (Hordeum vulgare L.) and the identification of clones containing putative resistance genes Theor Appl Genet 2000 101 1093 1099 10.1007/s001220051584

[B41] Eurofins MWG Biotech http://www.eurofinsdna.com

[B42] Meyer M Briggs AW Maricic T Höber B Höffner B Krause J Weihmann A Pääbo S Hofreiter M From micrograms to picograms: quantitative PCR reduces the material demands of high-throughput sequencing Nucleic Acids Res 2008 36 e5 10.1093/nar/gkm1095 18084031 PMC2248761

[B43] Ning Z Cox AJ Mullikin JC SSAHA: a fast search method for large DNA databases Genome Res 2001 11 1725 1729 10.1101/gr.194201 11591649 PMC311141

[B44] Szafranski K Jahn N Platzer M tuple_plot: fast pairwise nucleotide sequence comparison with noise suppression Bioinformatics 2006 22 1917 1918 10.1093/bioinformatics/btl277 16766553

