# Supplementary figures and images for: De novo 454 sequencing of barcoded BAC pools for comprehensive gene survey and genome analysis in the complex genome of barley
Source: BMC Genomics. 2009 Nov 20;10:547. doi: 10.1186/1471-2164-10-547 (PMC2784808; doi:10.1186/1471-2164-10-547)

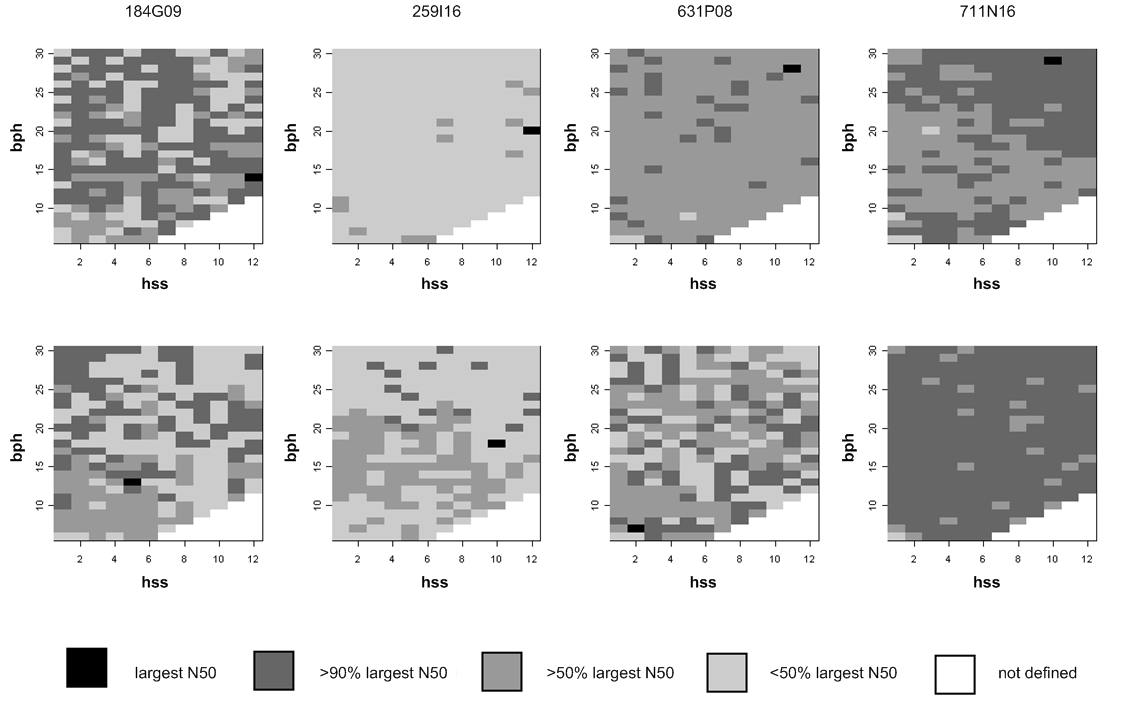

Supplement: Additional file 3 — Figure S1. Heat maps of N50 lengths of different assemblies. Heat maps visualizing the assembly results of 454 sequences of the four complete reference BACs (top: set 1; bottom: set 2) by MIRA under different combinations of hss (hash saving steps, X-axis) and bph (bases per hash, Y-axis). BACs from left to right are: 184G09, 259I16, 631P08, 711N16. Black fields indicate the hss/bph combinations resulting in the highest N50 values for the respective BAC. Dark to light gray fields mark values producing a contig with >90%, >50% and <50% of these values, respectively. White fields represent meaningless combinations (hss > bph). [file 1471-2164-10-547-S3.PNG]

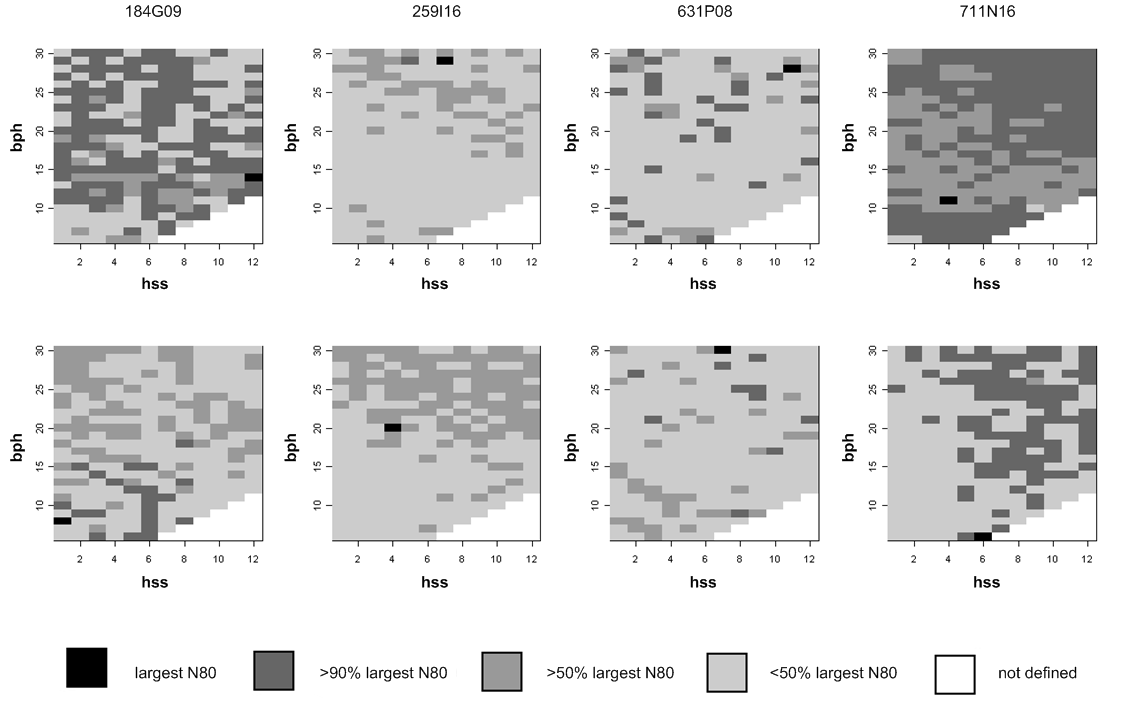

Supplement: Additional file 4 — Figure S2. Heat maps of N80 lengths of different assemblies. Heat maps visualizing the assembly results of 454 sequences of the four complete reference BACs (top: set 1; bottom: set 2) by MIRA under different combinations of hss (hash saving steps, X-axis) and bph (bases per hash, Y-axis). BACs from left to right are: 184G09, 259I16, 631P08, 711N16. Black fields indicate the hss/bph combinations resulting in the highest N80 values for the respective BAC. Dark to light gray fields mark values producing a contig with >90%, >50% and <50% of these values, respectively. White fields represent meaningless combinations (hss > bph). [file 1471-2164-10-547-S4.PNG]

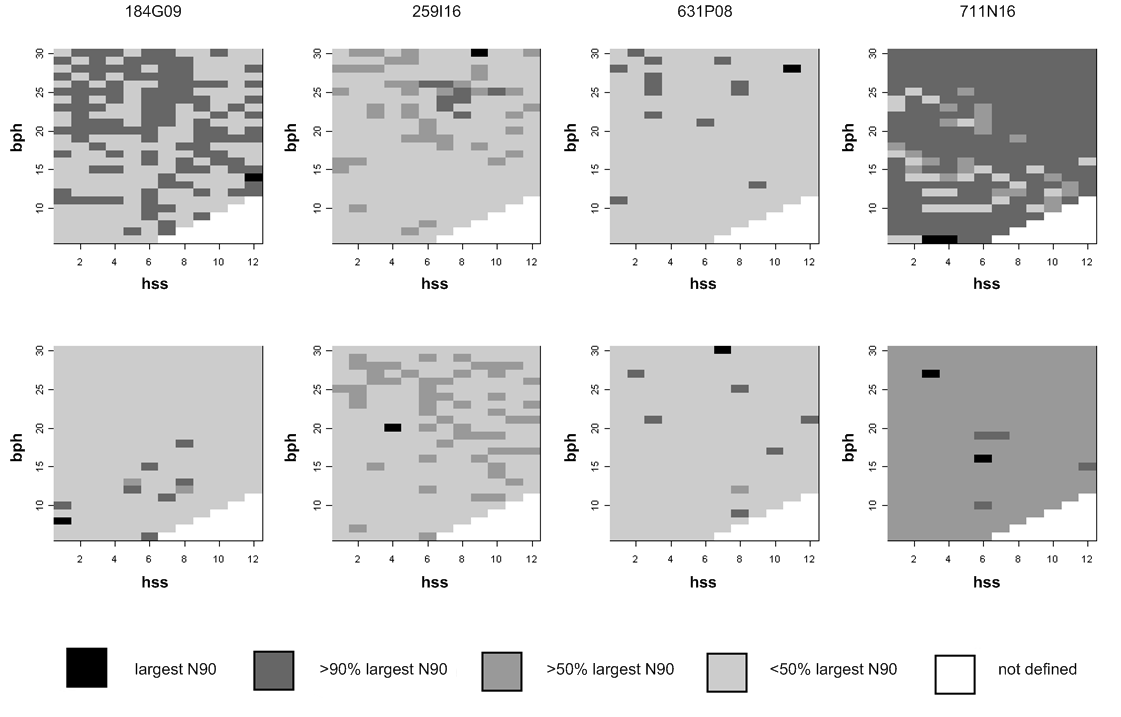

Supplement: Additional file 5 — Figure S3. Heat maps of N90 lengths of different assemblies. Heat maps visualizing the assembly results of 454 sequences of the four complete reference BACs (top: set 1; bottom: set 2) by MIRA under different combinations of hss (hash saving steps, X-axis) and bph (bases per hash, Y-axis). BACs from left to right are: 184G09, 259I16, 631P08, 711N16. Black fields indicate the hss/bph combinations resulting in the highest N90 values for the respective BAC. Dark to light gray fields mark values producing a contig with >90%, >50% and <50% of these values, respectively. White fields represent meaningless combinations (hss > bph). [file 1471-2164-10-547-S5.PNG]

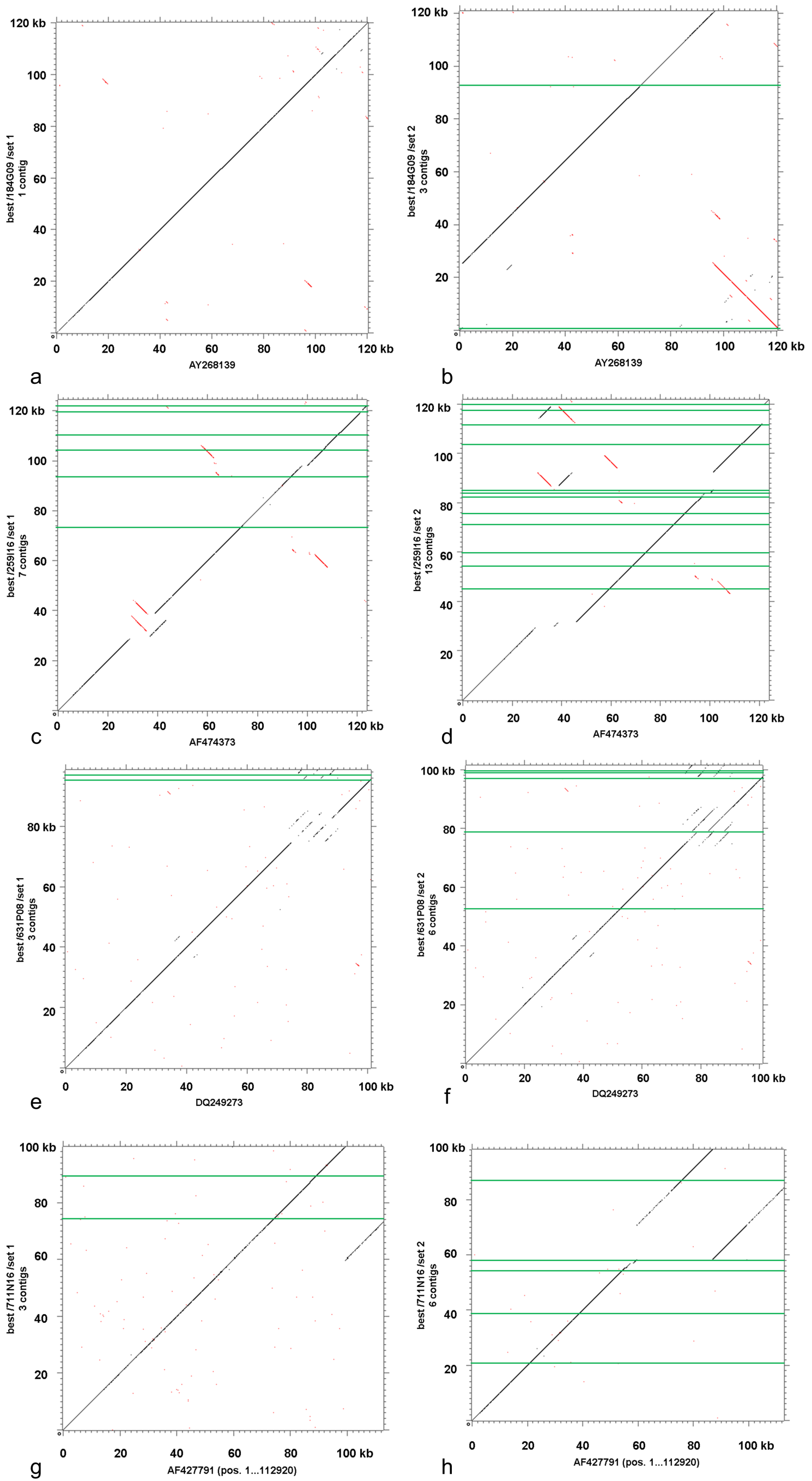

Supplement: Additional file 6 — Figure S4. Tupleplots of best MIRA assemblis versus Sanger reference sequence. Tupleplots show comparisons of best MIRA assembly contigs > 1 kb (y-axis) to the complete Sanger reference sequences (x-axis). a) 184G09 Set1/AY268139; b) 184G09 Set2/AY268139; c) 259I16 Set1/AF474373; d) 259I16 Set2/AF474373; e) 631P08 Set1/DQ249273; f) 631P08 Set2/DQ249273; g) 711N16 Set1/AF427791 (pos. 1...112.920); h) 711N16 Set2/AF427791 (pos. 1...112.920). [file 1471-2164-10-547-S6.PNG]

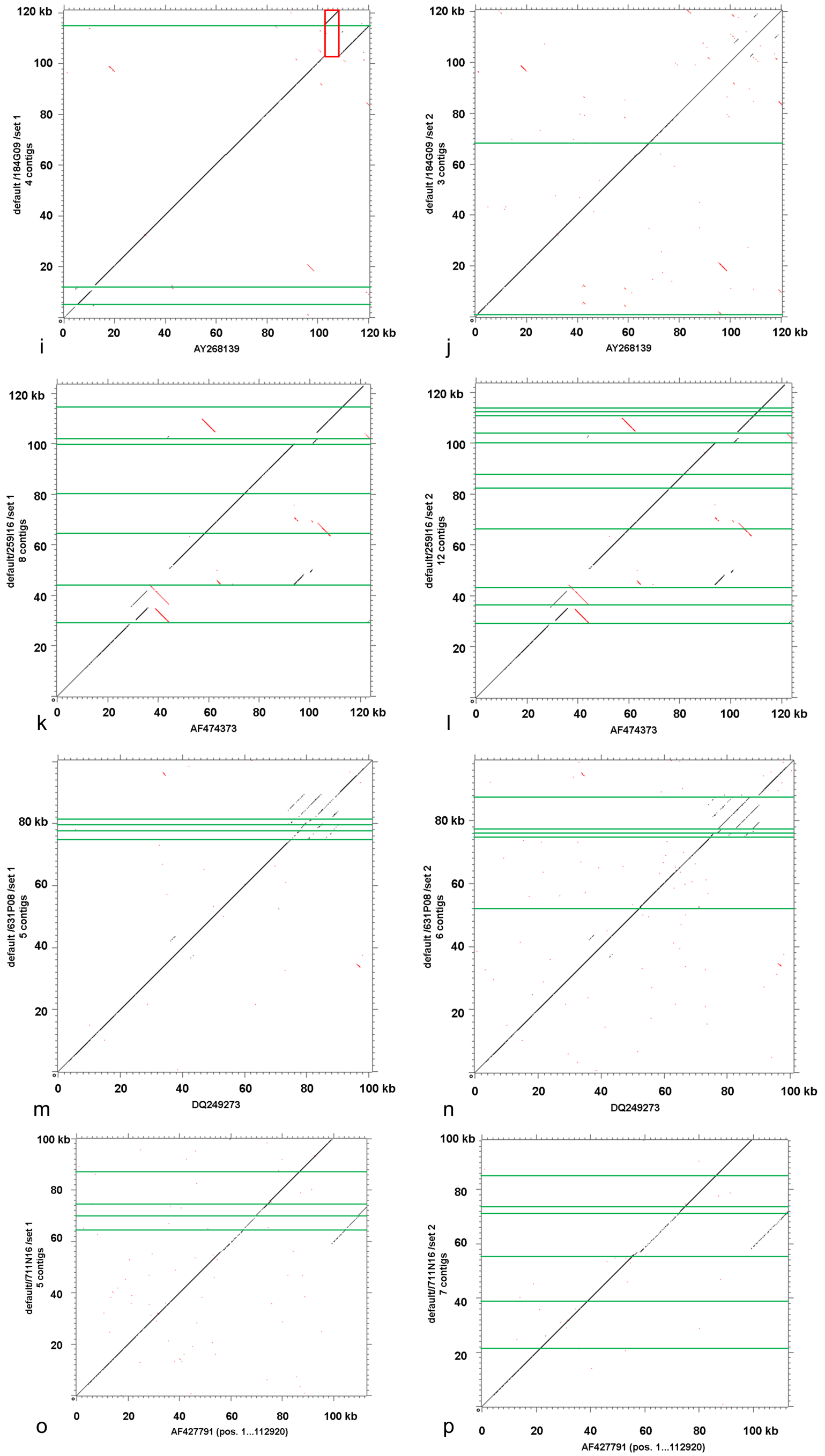

Supplement: Additional file 7 — Figure S5. Tupleplots of default MIRA assemblis versus Sanger reference sequence. Tupleplots show comparisons of best MIRA assembly contigs > 1 kb (y-axis) to the complete Sanger reference sequences (x-axis). i) 184G09 Set1/AY268139; j) 184G09 Set2/AY268139; k) 259I16 Set1/AF474373; l) 259I16 Set2/AF474373; m) 631P08 Set1/DQ249273; n) 631P08 Set2/DQ249273; o) 711N16 Set1/AF427791 (pos. 1...112.920); p) 711N16 Set2/AF427791 (pos. 1...112.920). [file 1471-2164-10-547-S7.PNG]

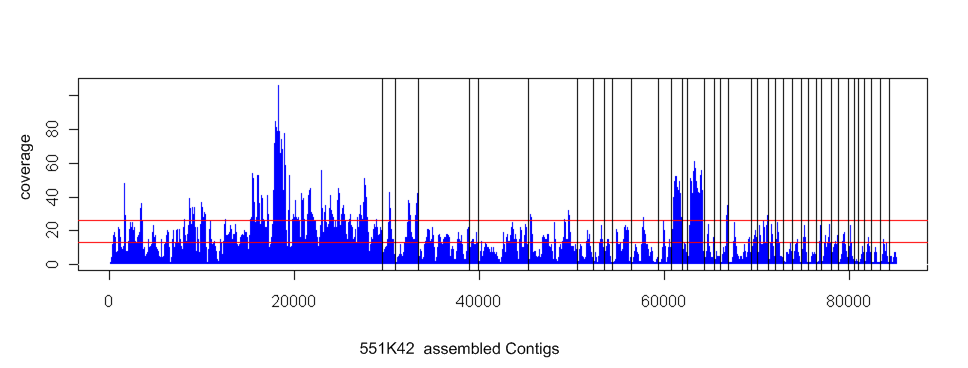

Supplement: Additional file 10 — Figure S6. Coverage Diagram of BAC 551K24. Coverage of the best MIRA assembly contigs by 454 reads from BAC 551K24, set1. Black vertical lines separate the contigs; red horizontal lines indicate the median and the twofold median, respectively. [file 1471-2164-10-547-S10.PNG]

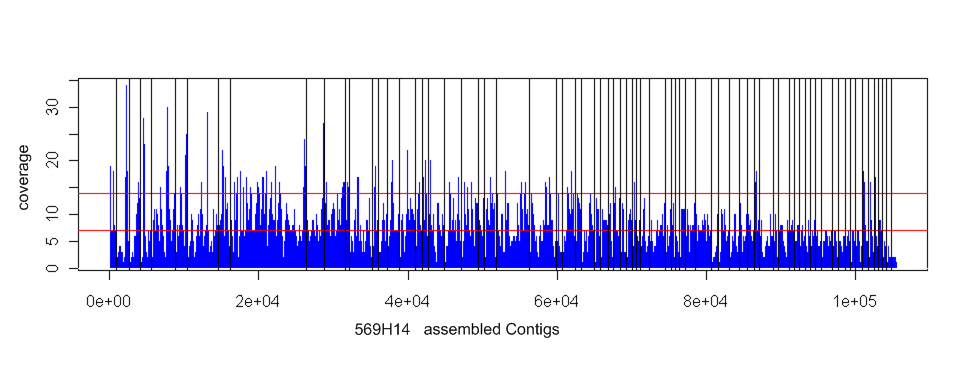

Supplement: Additional file 11 — Figure S5. Coverage Diagram of BAC 569H14. Coverage of the best MIRA assembly contigs by 454 reads from BAC 569H14, set1. Black vertical lines separate the contigs; red horizontal lines indicate the median and the twofold median, respectively. [file 1471-2164-10-547-S11.PNG]
